# Colistin Resistance Among Multiple Sequence Types of *Klebsiella pneumoniae* Is Associated With Diverse Resistance Mechanisms: A Report From India

**DOI:** 10.3389/fmicb.2021.609840

**Published:** 2021-02-22

**Authors:** Mudsser Azam, Rajni Gaind, Gulshan Yadav, Amit Sharma, Kirti Upmanyu, Manisha Jain, Ruchi Singh

**Affiliations:** ^1^ICMR-National Institute of Pathology, New Delhi, India; ^2^Department of Microbiology, VMMC and Safdarjung Hospital, New Delhi, India

**Keywords:** colistin resistance, *Klebsiella pneumoniae*, *phoPQ*, *pmrAB*, carbapenem resistance

## Abstract

**Background:** The resistance to colistin and carbapenems in *Klebsiella pneumoniae* infections have been associated with increased morbidity and mortality worldwide. A retrospective observational study was conducted to determine the prevalence and molecular events contributing to colistin resistance.

**Methods:** Clinical samples were screened for colistin resistance and underlying mechanisms were studied by PCR-based amplification and sequence analysis of genes of two-component regulatory system (*phoPQ* and *pmrAB*), regulatory transmembrane protein-coding *mgrB*, and mobilized colistin resistance genes (*mcr-1-8*). Gene expression of *pmrC* and *pmrK* was analyzed by qRT-PCR, and the genetic relationship was assessed by MLST. The putative effect of amino-acid substitutions was predicted by a combination of bioinformatics tools.

**Results:** Of 335 *Klebsiella* spp. screened, 11 (3.2%) were identified as colistin-resistant (MIC range, 8 to >128 μg/ml). *K. pneumoniae* isolates belonged to clonal complex-11 (CC11) with sequence types (STs): 14, 16, 43, 54, 147 and 395, whereby four isolates conferred three novel STs (3986, 3987 and 3988) profiles. Sequence analysis revealed non-synonymous potentially deleterious mutations in *phoP* (T151A), *phoQ* (del87–90, del263–264, L30Q, and A351D), *pmrA* (G53S), *pmrB* (D150V, T157P, L237R, G250C, A252G, R315P, and Q331H), and *mgrB* (C28G) genes. The *mgrB* gene in three strains was disrupted by insertion sequences encoding IS*1*-like and IS*5*/IS*1182* family-like transposase genes. All 11 isolates showed an elevation in the transcription level of *pmrC* gene. Mobilized colistin-resistance (*mcr*) genes were not detected. All but one of the colistin-resistant isolates was also resistant to carbapenems; β-lactamase genes *bla_NDM-1-like_*, *bla_OXA-48-like_*, and *bla_CTX-M-like_* were detected in eight, five, and nine isolates, respectively.

**Conclusion:** All the studied colistin- and carbapenem-resistant *K. pneumoniae* isolates were genetically distinct, and various mechanisms of colistin resistance were detected, indicating its spontaneous emergence in this bacterial species.

## Introduction

*Klebsiella pneumoniae*, a nosocomial pathogen, accounts for one-third of worldwide reported Gram-negative infections (Navon-Venezia et al., 2017). Multidrug-resistant (MDR) *K. pneumoniae* are resistant to extended-spectrum cephalosporins, carbapenems, aminoglycosides, and fluoroquinolones and present a significant challenge to the clinicians. Available data showcase a significant increase in *K. pneumoniae* in India’s carbapenem-resistant isolates from 29% in 2008 to 57% in 2016, limiting the treatment of life-threatening infections (Gandra et al., 2016; Dixit et al., 2019). The unavailability of new antimicrobial agents to combat carbapenem-resistant *K. pneumoniae* infections has revived the use of polymyxins (colistin and polymyxin B). However, the indiscriminate use of polymyxins in animals, aquaculture, and agriculture in the last decades has compounded the issue of drug resistance (Nguyen et al., 2016; Zhang et al., 2019).

Colistin-resistant *K. pneumoniae* exhibits a high degree of genetic plasticity where a point mutation and/or genetic disruption in two-component regulatory systems (TCRS), i.e., *pmrAB* and *phoPQ*, are known to confer polymyxin resistance (Cannatelli et al., 2014a; Jayol et al., 2014). Besides, polymyxin resistance is often observed by the inactivation of *mgrB*, a regulatory trans-membrane protein that controls the kinase activity of *phoQ* in *phoPQ* TCRS, by point mutations, indels, or insertion sequences (IS*5-like*, IS*1F*, IS*Kpn13*, IS*Kpn14*, IS*10R*; Cannatelli et al., 2015; Aires et al., 2016). The spread of colistin resistance has accelerated by acquiring plasmid-encoded mobile colistin resistance (*mcr*)- genes. After the first report in China (November 2015), *mcr*-genes were detected in *E. coli*, *K. pneumoniae*, *Shigella sonnei*, *Salmonella enterica*, and many other bacterial strains from colonized and infected humans, food (meat and vegetables), farm and wild animals, and aquatic environments (Liu et al., 2016; Carroll et al., 2019; Zhang et al., 2019). Selective colistin pressure in different sectors has induced constant emergence and evolution of *mcr*-genes, and *mcr-1* to *mcr-10* genes with multiple variants have been identified in colistin-resistant bacteria isolated from several sources (Gharaibeh and Shatnawi, 2019; Wang et al., 2020).

Increasing incidences of colistin resistance among nosocomial *K. pneumoniae* isolates have been reported from Europe, Asia, North America, South America, and Africa (Rojas et al., 2017; Lomonaco et al., 2018; Boszczowski et al., 2019; Zafer et al., 2019). Many isolated case reports and outbreaks of MDR *K. pneumoniae* infections were reported from different parts of India (Goel et al., 2014; Bhaskar et al., 2017; Kaur et al., 2017; Mohanty et al., 2017; Pragasam et al., 2017; Aggarwal et al., 2018). High mortality rate (approximately 69%) in bloodstream infections due to carbapenem- and colistin-resistant *K. pneumoniae* was also noted among Indian patients (Kaur et al., 2017; Manohar et al., 2017; Pragasam et al., 2017; Jajoo et al., 2018; Palani et al., 2020).

The accretion in clinical isolates of colistin- and carbapenem-resistant *K. pneumoniae* warrants further investigation into the epidemiology and underlying molecular mechanisms. Detection of colistin resistance is a challenge in clinical diagnosis, and evolving breakpoints have complicated the problem. Furthermore, horizontal gene transfer and the spread of clones with resistance traits confront the therapeutic control in nosocomial settings (Diene and Rolain, 2014). This study aimed to determine the prevalence of colistin-resistant *K. pneumoniae* in a tertiary care hospital in India; their clonal relationship and the molecular events contributing to colistin resistance.

## Materials and Methods

### Bacterial Strains

Clinical samples collected at Safdarjung Hospital and directed to the Department of microbiology for routine identification from August 2017 to January 2018 were included in the study. *Klebsiella* spp. were identified and screened for colistin resistance following standard broth microdilution method using cation-adjusted Mueller Hinton Broth (HiMedia, India) with EUCAST interpretation (S: ≤2 mg/L, R: >2 mg/L) guidelines (2018). Isolates were identified by a VITEK 2 GN card and confirmed by 16S rRNA gene sequence analysis (Azam et al., 2016). Ethics clearance was obtained from the institutional ethics committee: (i) ICMR-National Institute of Pathology, New Delhi, India (IEC No: NIP-IEC/2/3/17/06) and (ii) VMMC and Safdarjung Hospital, New Delhi, India (IEC No: IEC/VMMC/SJH/Project/1028). The demographic and clinical details of the patients were obtained from electronic medical records available in the hospital intranet.

### Antimicrobial Susceptibility Tests

Colistin-resistant isolates were subjected to antibiotic susceptibility testing by Kirby-Bauer disc diffusion method on Mueller Hinton Agar plates (HiMedia, India) against the ampicillin, ampicillin/clavulanic acid, amikacin, azithromycin, cefotaxime, chloramphenicol, ciprofloxacin, gentamicin, ertapenem, imipenem, tazobactam/piperacillin, tetracycline, and trimethoprim.

Minimum inhibitory concentrations (MICs) were determined by *E*-test (trimethoprim-sulfamethoxazole, amoxicillin-clavulanic acid, and imipenem) and broth microdilution (chloramphenicol, ciprofloxacin, colistin, gentamicin, kanamycin, polymyxin B, rifampicin, nalidixic acid, tigecycline, and tetracycline). *E. coli* ATCC 25922 (antibiotic-susceptible), *K. pneumoniae* ATCC 700603 (ESBL-producing), and *E. coli* NCTC 13846 (colistin-resistant) isolates were used as quality control strains. Results were interpreted as per CLSI (2018) guidelines and for tigecycline, EUCAST (2018) guidelines were followed. The susceptibility profile of *K. pneumoniae* isolates was determined using the WHONET (v20.8.21) database software.

### Multilocus Sequence Typing

Multilocus sequence typing (MLST) for seven housekeeping genes, *rpoB*, *gapA*, *mdh*, *pgi*, *phoE*, *infB*, and *tonB*, was performed using the Pasteur institute MLST scheme (Diancourt et al., 2005). A total of 3,550 MLST profiles of *K. pneumoniae* available till 2nd March 2019, were extracted from Pasteur institute MLST database.^1^ Sequence types (STs) were determined by comparing the sequence of isolates against *K. pneumoniae* MLST database (Feil et al., 2004). Novel ST Profiles were submitted to the curator for assignment of new sequence type. Clustering and comparative analysis of related STs were performed using default conservative definition of sharing six of the seven loci of eBURST version 3 software.

### Molecular Characterization of Genes Contributing to Colistin and β-Lactam Resistance

Lipid A modifying genes, i.e., *phoP*, *phoQ*, *pmrA*, *pmrB*, and *mgrB*, associated with colistin resistance were amplified and sequenced using gene-specific primers designed using the external region of the gene sequence (Supplementary Table S1). To determine the presence of plasmid-encoded colistin resistance, gene-specific primers were used to amplify *mcr-1-8* genes. *K. pneumoniae* isolates were also analyzed for the presence of the ESBL gene (*bla_CTX-M-like_*) and carbapenemase genes (*bla_KPC-like_*, *bla_NDM-1-like_*, and *bla_OXA-48-like_*), conferring resistance to a broad range of β-lactam antibiotics (primer sequence information used for the amplification of specific genes is given in Supplementary Table S1). Genomic DNA from *K. pneumoniae* ATCC 700603 (colistin-susceptible), *E. coli* NCTC 13846 (*mcr-1* positive), *K. pneumoniae* ATCC BAA1705 (*bla_KPC_* positive), *K. pneumoniae* ATCC BAA2156 (*bla_NDM_* positive), and *E. coli* MRE2 (*bla_CTX-M_* positive, GenBank accession # KM873162) were used as control strains.

### Gene Expression Analysis by qRT-PCR

The expression level of *pmrK* (encoding L-Ara4N transferase) and *pmrC* (encoding PEtN transferase) genes were analyzed using gene-specific primers by qRT-PCR in colistin treated and untreated samples. Colistin-resistant isolates and *K. pneumoniae* ATCC 700603 were grown to the mid-log phase in cation-adjusted Mueller Hinton Broth supplemented with and without colistin sulphate (4 μg/ml; Sigma). Bacterial cells were harvested, and total RNA was extracted using RNeasy kit (Qiagen) and treated with DNaseI. cDNA synthesized from 1 μg of total RNA using RevertAid first-strand cDNA synthesis kit (Thermo Scientific) was subjected to Real-time PCR amplification in 25 μl reactions (in triplicates) containing 10 pmoL forward and reverse primers and 1x Fast SYBR green PCR master mix (Applied BioSystems) under the following conditions: 1 cycle of 95°C for 20 s, 40 cycles of 95°C for 3 s, and 60°C for 30 s. After each run, melt curve analysis was performed to ensure single amplicon production, under the conditions 95°C for 15 s, 60°C for 1 min, and 95°C for 15 s. Relative gene expression levels were calculated using the 2^ΔΔCT^ formula, and ≥2 fold change in the expression (relative to wild type) was considered the increase in expression and below ≤0.5 as repression. The *rpsL* gene (encoding ribosomal protein) was used as an internal control. Colistin-susceptible *K. pneumoniae* ATCC 700603 was used for normalization. The student’s paired “*t*” test was performed for calculating the significance of differences observed in the expression levels under colistin untreated and treated conditions with the help of GraphPad Prism software version 8.0 (GraphPad software inc. CA, United States).

### Analysis of Deleterious Substitution

The amino acid substitutions in the genes (*mgrB*, *phoP*, *phoQ*, *pmrA*, and *pmrB*) associated with the colistin resistance phenotype in clinical isolates were defined by comparing the query sequence against *K. pneumoniae* ATCC 700603. To predict the phenotypic effect of these substitutions on protein structure and function, these were analyzed by three different bioinformatics tools: sorting intolerant from tolerant (SIFT), polymorphism phenotyping (PolyPhen-2), and protein variation effect analyzer (PROVEAN) with default parameters (Ng and Henikoff, 2003; Adzhubei et al., 2010; Sim et al., 2012; Choi and Chan, 2015). The mutations found to be deleterious by at least two of the three analysis software were considered potentially deleterious, while mutations with low confidence intervals were treated as neutral.

### Nucleotide Sequence Accession Numbers

The nucleotide sequences of colistin-resistant *K. pneumoniae* isolates under study were deposited at a GenBank nucleotide sequence database under the following accession numbers: *16S rRNA* gene (MH411220, MH410611, MH411072–MH411080), *phoP* gene (MH424384–MH424392, MH450205, MH450206), *phoQ* gene (MH424393–MH424402, MH450207), *pmrA* gene (MH450188–MH450197), and *pmrB* gene (MH450198–MH450204, MH688167–MH688170). Nucleotide sequences of the *mgrB* genes have been deposited under GenBank accession numbers: wild type *mgrB* gene (MH424403–MH424409, MH671333) and mutated/disrupted *mgrB* gene (MK625062–MK625064).

## Results

**Figure 1:**
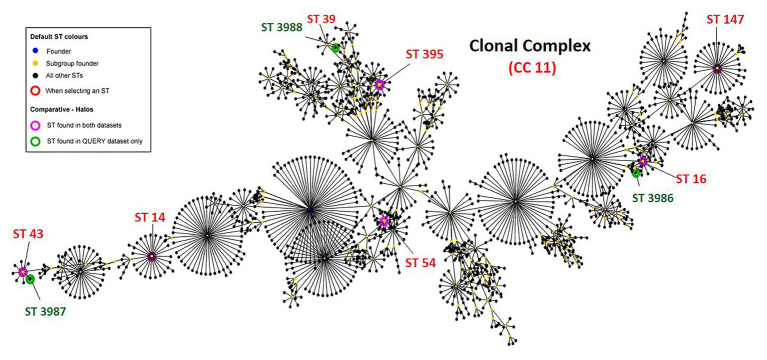
Clonal complex CC11 with 1,562 sequence types. Green halos represents novel sequence types found in this study and submitted to curator of *Klebsiella pneumoniae* MLST database Pasteur institute (https://bigsdb.pasteur.fr/klebsiella/klebsiella.html), pink halos represent isolates with defined sequence types. *K. pneumoniae* ATCC 700603 is ST489 and designated as singleton by eBURST v3 and hence not shown here.

A total of 335 *K. pneumoniae* were isolated from urine (*n* = 153), blood (*n* = 20), pus (*n* = 85), tracheal aspirate (*n* = 9), wound (*n* = 7), and other specimens (*n* = 61) within a period of 6 months (2017–2018) in the hospital. Of the total, 11 (3.2%) *Klebsiella* isolates representing one isolate per patient from the clinical samples viz. urine (5), pus (3), tissue (1), wound swab (1), and blood (1) were identified to be colistin-resistant. All 11 patients had a hospital stay for more than 10 days and encountered invasive devices like intravenous lines, ventilators, or catheters. These patients received colistin during or before their sample collection for microbiology culture analysis. Molecular identification confirmed the isolates as *K. pneumoniae* and MLST analysis revealed the sequence types ST14 (*n* = 1), ST16 (*n* = 1), ST43 (*n* = 1), ST54 (*n* = 1), ST147 (*n* = 1), and ST395 (*n* = 2; Table 1). Four of the isolates belonged to three novel ST profiles and were assigned new sequence type numbers as ST3986 (MRK1), ST3987 (MRK5 and MRK6), and ST3988 (MRK10). Comparative and clustering analysis by eBURST v3 of query dataset with a total of 3,550 already reported MLST profiles of *K. pneumoniae* suggested that all 11 isolates of the query dataset belonged to the most prominent and diverse clonal complex 11 (CC11). The novel sequence types ST3986, ST3987, and ST3988 had evolutionarily related sub-founders as ST16, ST43, ST39, respectively (Figure 1). The reference strain *K. pneumoniae* ATCC 700603 used in this study belonged to ST489 and was designated as a singleton by eBURST v3.

**Table 1:** Demographic, clinical, and microbiological features of 11 colistin-resistant *K. pneumoniae* isolates.

Bacterial isolate	Source, year of isolation	Hospital ward	Sequence type	Carbapenemase genes(*bla_NDM-1_*, *bla_OXA-48_*)	ESBL gene (*bla_CTX-M-like_*)	Non-susceptibility to antibiotics by disc diffusion assay	Susceptibility profile^b^	ICU stay, outcome
MRK1	Urine, 2017	-	ST3986^a^	*bla_NDM-1_*	*bla_CTX-M-like_*	AMP, AMC, AMK, CIP, CTX, ETP, GEN, IPM, TMP, AZM, TET, TZP	PDR	no, recovered
MRK2	Pus, 2017	Surgical ICU	ST43	*bla_OXA-48_*	*bla_CTX-M-like_*	AMP, AMC, AMK, CIP, CTX, ETP, GEN, IPM, TZP	MDR	no, recovered
MRK3	Urine, 2017	Rehabilitation	ST54	*bla_NDM-1_*	*bla_CTX-M-like_*	AMP, AMC, AMK, AZM, CIP, CTX, ETP, IPM, GEN, IPM,TMP, TZP	XDR	no, recovered
MRK4	Tissue, 2017	-	ST16	*bla_NDM-1_*	*bla_CTX-M-like_*	AMP, AMC, AMK, AZM, CIP, CTX, ETP, GEN, IPM, TMP, TET, TZP	PDR	yes, death
MRK5	Urine, 2017	Orthopedics	ST3987^a^	*bla_OXA-48_*	*-*	AMP, AMC, AMK, AZM, CIP, CTX, GEN, TZP	XDR	no, recovered
MRK6	Blood, 2017	Medical ICU	ST3987^a^	*bla_NDM-1_*, *bla_OXA-48_*	*bla_CTX-M-like_*	AMP, AMC, AMK, AZM, CIP, CTX, ETP, GEN, IPM, TMP, TZP	XDR	yes, death
MRK7	Urine, 2017	-	ST395	*bla_OXA-48_*	*bla_CTX-M-like_*	AMP, AMC, AZM, CIP, CHL, CTX, ETP, GEN, IPM, TMP, TZP	XDR	no, recovered
MRK8	Wound Swab, 2018	Burns	ST395	*bla_NDM-1_*	*bla_CTX-M-like_*	AMP, AMC, AMK, CIP, CTX, ETP, GEN, IPM, TET, TMP, TZP	PDR	yes, death
MRK9	Urine, 2018	Obstetrics and gynecology	ST14	*bla_NDM-1_*	*bla_CTX-M-like_*	AMP, AMC, AMK, AZM, CIP, CHL, CTX, ETP, GEN, IPM, GEN, TMP	XDR	no, recovered
MRK10	Pus, 2018	Burns	ST3988^a^	*bla_NDM-1_*, *bla_OXA-48_*	*-*	AMP, AMC, AMK, AZM, CIP, CTX, ETP, GEN, IPM, TMP, TET, TZP	PDR	yes, death
MRK11	Pus, 2018	Medical ICU	ST147	*bla_NDM-1_*	*bla_CTX-M-like_*	AMP, AMC, AMK, CIP, CTX, ETP, GEN, IPM, TMP, AZM, TET, TZP	XDR	yes, recovered

Three-letter abbreviation code and amount of antibiotic (μg) in the disc: AMP, ampicillin (10 μg); AMC, ampicillin/clavulanic acid (20/10 μg); AMK, amikacin (30 μg); AZM, azithromycin (15 μg); CTX, cefotaxime (30 μg); CHL, chloramphenicol (30 μg); CIP, ciprofloxacin (5 μg); GEN, gentamicin (10 μg); ETP, ertapenem (10 μg); IPM, imipenem (10 μg); TZP, tazobactam/piparacillin (100/10 μg); TET, tetracycline (30 μg); and TMP, trimethoprim (5 μg).

a Novel sequence type identified in this study. MLST analysis was performed following Pasteur institute MLST database (https://bigsdb.pasteur.fr/klebsiella/klebsiella.html).

b Susceptibility profile as determined by WHONET (v20.8.21) database software. MDR: multidrug resistant; PDR- pan-drug resistant, XDR- extensively drug resistant.

### Antimicrobial Susceptibility Testing

All 11 tested *K. pneumoniae* isolates were resistant to cephalosporins, carbapenems, fluoroquinolones, aminoglycosides, macrolides, and trimethoprim by disc diffusion method (Table 1). Three *K. pneumoniae* isolates (MRK1, MRK9, and MRK10) were resistant to all antibiotics tested by disc diffusion assay except with intermediate resistant phenotype to chloramphenicol (MRK1 and MRK10) and tetracycline (MRK9), as per CLSI standards. MIC-values for colistin and polymyxin B ranged from 8 to >128 μg/ml and 16 to >64 μg/ml, respectively. Unexpectedly, MICs of 5 (45.5%) isolates to colistin were ≥128 μg/ml (Table 2). MICs of amoxicillin/clavulanic acid and imipenem were >256 μg/ml and ≥8 μg/ml, respectively, for the 11 tested isolates except for MRK5 (4 μg/ml and 2 μg/ml). High MIC values were noted for kanamycin (≥256 μg/ml; *n* = 10), gentamycin (≥256 μg/ml; *n* = 6), co-trimoxazole (>32 μg/ml; *n* = 9), and tetracycline (>128 μg/ml; *n* = 5) among colistin-resistant *K. pneumoniae*.

**Table 2:** Minimum inhibitory concentration (MIC) in μg/ml of different antibiotics tested by *E*-test and broth microdilution method for 11 *K. pneumoniae* isolates.

Antibiotic name	Breakpoints	%R	%I	%S	MIC50	MIC90	MIC range
Amoxicillin/Clavulanic acid^*^	S ≤ 8 R ≥ 32	90.9	0	9.1	>256	>256	4 to >256
Imipenem^*^	S ≤ 1 R ≥ 4	90.9	9.1	0	24	32	2 to >256
Gentamicin	S ≤ 4 R ≥ 16	100	0	0	256	>256	128 to >256
Kanamycin	S ≤ 16 R ≥ 64	100	0	0	256	>256	64 to >256
Rifampin	S ≤ 1 R ≥ 4	100	0	0	128	256	4 to >128
Nalidixic acid	S ≤ 16 R ≥ 32	100	0	0	256	>256	64 to >256
Ciprofloxacin	S ≤ 1 R ≥ 4	100	0	0	8	32	8 to >256
Trimethoprim/Sulfamethoxazole^*^	S ≤ 2 R ≥ 4	81.8	0	18.2	64	64	0.064 to >32
Colistin	S ≤ 2 R ≥ 8	100	0	0	64	256	8 to >128
Polymyxin B	S ≤ 2 R ≥ 8	100	0	0	64	128	16 to >64
Chloramphenicol	S ≤ 8 R ≥ 32	63.6	18.2	18.2	32	256	4 to 256
Tetracycline	S ≤ 4 R ≥ 16	54.5	0	45.5	16	256	1 to >128
Tigecycline	S ≤ 1 R ≥ 4	63.6	9.09	27.2	0.5	1.5	0.25 to >256

Susceptibility results were interpreted as per CLSI (2018) guidelines and for tigecycline EUCAST (2018) guidelines were followed. R, resistant; I, intermediate; S, susceptible.

* *E*-test was performed to determine the MIC values.

### Molecular Determinants for Drug Resistance

Sequence analysis of Lipid A modifying genes of all the 11 isolates of *K. pneumoniae* revealed non-synonymous nucleotide mutations at two different positions of the *phoP* gene, 18 positions of the *phoQ* gene, nine positions of the *pmrA* gene, 37 positions of the *pmrB* gene, and two positions of the *mgrB* gene. Of these mutations, one in *phoP*, four in *phoQ*, one in *pmrA*, eight in *pmrB*, and one in *mgrB* were found to be potentially deleterious (Table 3). The protein sequence of *phoP* genes of all colistin-resistant *K. pneumoniae* isolates showed no difference with the wild type strain (*K. pneumoniae* ATCC 700603) except for MRK9 strain that exhibited T151A substitution. *phoQ* gene was mutated in two strains, i.e., MRK8 (L30Q) and MRK11(A351D), whereas, MRK5 and MRK6 isolates exhibited deletion of four and two amino acids at 87–90 and 267–268 positions, respectively. All other isolates exhibited neutral changes compared to sensitive strain at *phoQ* locus.

**Table 3:** List of amino acid substitutions or disruptions among *mgrB*, *phoP*, *phoQ*, *pmrA*, and *pmrB* proteins among 11 colistin-resistant *K. pneumoniae* isolates.

Bacterial strain	Colistin MIC (μg/ml)	Amino acid substitutions^#^				
		*mgrB*	*phoP*	*phoQ*	*pmrA*	*pmrB*
MRK1	128	IS Insertion (IS*1*-like element, position 116–117)	-	-	-	G250C A252G
MRK2	64	-	-	-	-	D150V
MRK3	>128	IS Insertion (IS*5*/IS*1182*-like element, position 94–95)	-	-	-	-
MRK4	>128	IS Insertion (IS*1*-like element, position 116–117)	-	-	-	G250C
MRK5	128	-	-	Deletion (87–90)	-	L237R G250C A252G H267P R315P Q331H
MRK6	64	-	-	Deletion (267–268)	-	A252G
MRK7	32	-	-	-	-	- H267P
MRK8	8	-	-	L30Q	-	-
MRK9	32	C28G	T151A	-	-	T157P
MRK10	16	-	-	-	G53S -	-
MRK11	8	-	-	A351D	-	-

-, no deleterious mutation observed.

# SIFT, PolyPhen-2, and PROVEAN were used to determine the nature (neutral or deleterious) of amino acid substitutions. Mutations that were found to be potentially deleterious with at least two of the three software’s used were taken into account. Mutations showing low confidence intervals were treated as neutral.

The insertional inactivation of the *mgrB* gene was observed in three isolates, i.e., MRK1, MRK3, and MRK4. IS*1* family transposase was observed in *K. pneumoniae* MRK1 strain. In *K. pneumoniae* MRK3, *mgrB* gene was interrupted with a 1,066 bp fragment composed of the IS*5*/IS*1182* family transposase gene, non-coding sequence, and nine base pair inverted repeats (ACCAGGATG). An insertion sequence of 768 base pairs comprising IS*1* family transposase and a non-coding fragment was observed interrupting the *mgrB* gene of *K. pneumoniae* MRK4 strain. A base change T to G at position 82, leading to an amino acid substitution of C28G being observed in the *mgrB* gene of *K. pneumoniae* MRK9, which was found to be deleterious.

Analysis of *pmrA/pmrB* genes regulating the expression of *arnBCADTEF* operon decorating LPS showed amino acid changes at five positions in *pmrA* gene of four different isolates, none being deleterious except for MRK-10 exhibiting G53S. The *pmrB* gene was mutated in most isolates with a varying number of potentially deleterious mutations ranging from one mutation in MRK2, MRK6 and MRK9 to six deleterious mutations in MRK5 strain. In total, 23 neutral changes and eight potentially deleterious changes were observed in the *pmrB* gene. L30Q substitution of *phoQ* and D150V substitution of *pmrB* were observed to be highly intolerant under PROVEAN, SIFT, and PolyPhen-2 scoring criteria. Plasmid-encoded colistin resistance genes (*mcr-1-8*) did not amplify in any of the tested colistin-resistant *K. pneumoniae* isolates.

Colistin-resistant *K. pneumoniae* isolates resistant to carbapenems showed positive amplification of *bla_NDM-1-like_* and *bla_OXA-48-like_* gene in eight and five isolates, respectively. Furthermore, ESBL encoding *bla_CTX-M-like_* gene was found in six isolates, and one of the isolates, MRK6, showed positive amplification for three β-lactamase (*bla_NDM-1-like_*, *bla_OXA-48-like_*, and *bla_CTX-M-like_*) genes (Table 1).

### Expression of *pmrK* and *pmrC* Genes

**Figure 2:**
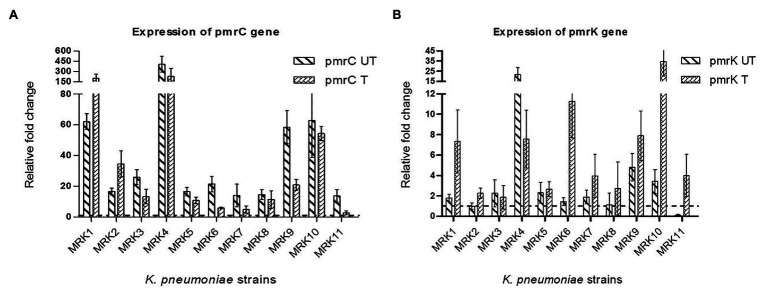
Fold changes ±SD in the expression of *pmrC* and *pmrK* genes among colistin-resistant *K. pneumoniae* isolates (*n* = 11) with respect to colistin-susceptible *K. pneumoniae* ATCC 700603 isolate are represented here. The *rpsl* gene was used as internal control. All the reactions (both under colistin treated and untreated conditions) were normalized using colistin-susceptible *K. pneumoniae* ATCC 700603 (colistin untreated- wild type). **(A)** relative fold-change of *pmrC* gene expression under colistin treated (*pmrC* T) and untreated conditions (*pmrC* UT); **(B)** relative fold-change of *pmrK* gene expression under colistin treated (*pmrK* T) and untreated conditions (*pmrK* UT). Values given are mean ± SD of three different experiments with qRT-PCR reactions performed in triplicate.

The fold change in the expression level of *pmrC* and *pmrK* genes is given in Figure 2. All the colistin-resistant *K. pneumoniae* isolates exhibited increased transcript levels of *pmrC* gene both in the presence or absence of colistin sulphate. The fold change in *pmrC* expression ranged from 2.7 ± 1.29 to 226.37 ± 118.63 when bacteria was subcultured with 4 μg/ml of colistin sulphate and 13.76 ± 4.091 to 404.71 ± 116.25-fold under untreated conditions. MRK4, MRK8, and MRK10 exhibited a comparable level of *pmrC* expression in both treated and untreated conditions (*p* = >0.05) whereas two isolates (MRK1 and MRK2) showed more than 2-fold increase in expression (*p* < 0.05) upon colistin treatment, and the remaining six isolates exhibited a decrease in *pmrC* expression from 1.5- to 5-fold (*p* < 0.05) upon colistin treatment. The expression level of *pmrK* gene under untreated condition was comparable to colistin-susceptible isolate in seven isolates (MRK1, MRK2, MRK3, MRK5, MRK6, MRK7, and MRK8), high (3.42 ± 1.171 to 21.67 ± 6.87 fold) in three isolates and down in isolate MRK11. Upon colistin treatment, the *pmrK* expression increased by 1.63- to 36.9-fold in seven isolates (MRK1, MRK2, MRK6, MRK7, MRK9, MRK10, and MRK11; *p* < 0.05) and was comparable in three isolates except for MRK4 where it decreased approximately 3-fold.

## Discussion

The rise in carbapenem-resistant *K. pneumoniae* isolates has enforced the increased application of polymyxin based therapies (mono and combination), ensuing in the development of colistin- and carbapenem-resistant *K. pneumoniae* strains. Reports of colistin resistance are emerging from different countries (Monaco et al., 2014; Rojas et al., 2017; Boszczowski et al., 2019). Our data showed 3.2% (11/335) of *K. pneumoniae* as colistin-resistant with minimal clonal relatedness. All 11 *K. pneumoniae* isolates belonged to same most prominent and diverse clonal complex-11 with sequence types (STs): 14, 16, 43, 54, 147, and 395, whereby four isolates conferred three novel ST (3986, 3987, and 3988) profiles. Although isolates MRK5 and MRK6 belonged to ST3987 and isolates MRK7 and MRK8 belonged to ST395, all four were isolated from patients of different wards of the hospital. The PubMed search for published literature identified limited studies describing the molecular mechanism of colistin resistance among *K. pneumoniae* isolates in India and abroad (Table 4). A thorough analysis showed that compared to other global studies, *K. pneumoniae* of very different STs conferred colistin resistance in India. However, Mavroidi et al. (2016) demonstrated colistin resistance due to clonal spread of KPC-producing *K. pneumoniae* belonging to clonal complex CC258 in Greece. Moubareck et al. (2018) showed 59% of colistin resistance in carbapenem-resistant *K. pneumoniae* was associated with locally prevalent ST14 clone in Dubai. High MIC values for colistin and carbapenems in *K. pneumoniae* causing bloodstream infections were observed in different studies from India (Ramesh et al., 2017; Aggarwal et al., 2018). *K. pneumoniae* resistant to both colistin and carbapenems have also been reported to be associated with increased risk of in-hospital mortality from India (Kaur et al., 2017; Aggarwal et al., 2018) and other parts of the world (Giacobbe et al., 2015; Rojas et al., 2017).

**Table 4:** Studies performed in India and abroad to understand the molecular mechanisms of colistin resistance among *K. pneumoniae* isolates.

Reference	Sequence Type (ST)^a^	WGS/targeted sequencing	Mutations observed in colistin-resistant isolates^b^			Transcript expression analysis of LPS modifying genes	*mcr-1 to 10* genes
			*phoPQ*	*mgrB*	*pmrAB*		
**Studies from India**							
This study (*n* = 11)	14,16, 43, 54, 147, 395, 3,986, 3,987, 3,988	Targeted sequencing	*phoP* (T151A) *phoQ* (L30Q, A351D), Deletion (87–90, 267–268)	*mgrB* (C28G) Insertion by IS*1*, IS*5*/IS*1182*-like elements	*pmrA* (Q140L^$^, G53S) *pmrB* (G250C, A252G,D150V, L237R, H267P, R315P, Q331H, R256G, T157P^#^)	*pmrC*-↑ *pmrK*-↑	negative
Palani et al. (2020) (*n* = 25)	-	Targeted sequencing	-	*mgrB* (C88T) Insertion by IS*Kpn14*, IS*Kpn26-*like elements, complete deletion	-	-	negative
Shankar et al. (2019a) (*n* = 1)	2,957	WGS	*phoP* (G273C) *phoQ* (P424L)	-	*pmrB* (A774T)	-	negative
Shankar et al. (2019b) (*n* = 19)	23, 147, 86, 11, 231, 14, 2096, 2,957	Targeted sequencing	*phoP* (A114R^@^, T151A^@^, E22K) *phoQ* (L209C^@^, W161L, G117D, V370E^@^, L172Q^@^, W182S^@^, V444F^@^, P424L, V446W^@^)	*mgrB* (C28G^@^, M1R^@^) Insertion by IS*903*, IS*Kpn14* and IS*Kpn26* elements	-	-	negative
Mathur et al. (2018) (*n* = 8)	11, 14, 231	WGS	*phoP* (R114A) *phoQ* (D150G)	*mgrB* (V1A, L24H)	*pmrB* (D150H, R256G^$^, L344P, T157P^#^, A246T)	-	negative
Pragasam et al. (2017) (*n* = 8)	14, 147, 231	WGS	*phoP* (R114A, R128A) *phoQ* (D146G, D150G)	*mgrB* -deletion of A at position 10, premature stop codon	*pmrB* (T157P^#^, A245T, R256G^$^, L344P)	-	negative
**Studies across the world**							
Cheong et al. (2019), Korea (*n* = 13)	11,461, 3,217	Targeted sequencing	*phoP* (R198H, K189D) *phoQ* (N152D, L414R)	-	*pmrA* (R203K) *pmrB* (N150D)	-	negative
Zhu et al. (2019), Greece (*n* = 8)	258, 147,	WGS	*phoP* (V53G^$^) *phoQ* (N253P, D438H, T439P).	*mgrB* insertion by IS*Kpn26*-like elements	*pmr*B (T140P^$^) *pmr*C (E307Stop^$^)	-	-
Zafer et al. (2019), Egypt (*n* = 22)	11, 101, 147, 16, 37, 383, 785, 1,399	Targeted sequencing	-	*mgrB* (P178Y)	-	-	*mcr-1* positive
Boszczowski et al. (2019), Brazil (*n* = 6)	11, 23, 340, and 437	WGS	*phoQ* (G150D)	*mgrB* (M1V, N25K, V26E, M27G, C28A, D29Y, I45R, P46L, W47F, N42L, K43D, F44P, I45P, P46S)	*pmrA* (T245A, R255G, P345L)	-	negative
Pitt et al. (2018), Brazil (*n* = 19)	147, 258, 11	WGS	*phoP* (A95S, P74L) *phoQ* (N253T, T281M^#^, G385C^#^, V446G)	*mgrB* (C28STOP^#^, Q30STOP^#^, D29E^#^) Insertion by IS*Kpn13*, IS*1*, IS*Kpn26*-like elements	*pmrB* (T140P, P158R^#^)	-	negative
Lomonaco et al. (2018), Pakistan (*n* = 10)	11, 14, 15, 101	WGS	-	Insertion by IS*Kpn25*, IS*1*, IS*5* elements	*pmrB* (T93P, N110T, T112P, T127P, T128P, L130P, L141P, V151G, T157P^#^, L159P, L164P, L213M, A246T, R256G^$^)	-	negative
Lu et al. (2018), West China (*n* = 5)	23, 412, 660 and 700	WGS	*-* *phoQ* (D150G)	-	*pmrB* (P344L)	-	*mcr-1* positive
Cain et al. (2018), UK (*n* = 1)	-	WGS	*phoQ* (K46Q^#^)	-	-	-	-
Leung et al. (2017), USA (*n* = 22)	17, 37, 258	WGS	-	*mgrB* (Q30R, Q30stop) Insertion by IS*Kpn26*-like, IS*903B*- like elements	*pmrB* (S85R, T157P^#^, H340R) *pmrF* (F280L, K322Q) *pmrJ* (E25A, R29K, I53V, L94I) *pmrK* (I117V, H156Q, D441E)	-	-
Jaidane et al. (2018), Tunisia (*n* = 13)	101, 15, 11, 147, 392	WGS	-	*mgrB* (F28C^#^), insertion by IS*1*-like elements	*pmrA* (A217V) *pmrB* (T246A, R256G^$^, T157P^#^)	-	negative
Haeili et al. (2017), Iran (*n* = 20)	-	Targeted sequencing	-	*mgrB* (Q30stop^#^, C39stop^#^) Insertion by IS5-like and IS*1*-like elements	*pmrB* (A246T, L213M, R256G^$^)	*pmrC*-↑ *pmrK*-↑	negative
Halaby et al. (2016), Netherlands (*n* = 13)	43, 1,423	WGS	*phoQ* (A21S^#^)	Insertion by IS*3*-like and IS*Kpn14* like elements	-	-	-
Cheng et al. (2015), Taiwan (*n* = 26)	11, 15, 29, 48, 421	Targeted sequencing	*phoP* (V3F, S86L) *phoQ* (L26P^#^, A150G, V258F)	*mgrB* (Stop48Y^#^)	*pmrB* (R256G^$^)	*pmrH*-↑ *pmrK*-↑ *mgrB*-↓	-
Jayol et al. (2015), South Africa (*n* = 1)	-	Targeted sequencing	*phoP* (N191Y^#^)	-	-	*phoP*-↑ *phoQ*-↑ *pmrD*-↑ *pmrC*-no change, *pmrA*-no change, *pmrB*-no change, *pmrK*-↑	-

↑, Increase in transcript expression level; ↓, decrease in transcript expression level; -, not detected/analyzed; n, Number of colistin-resistant isolates analyzed for molecular mechanisms of resistance.

$ Mutations observed in colistin-susceptible as well as colistin-resistant isolates.

# Experimental evidence for their role in imparting colistin resistance.

@ Deleterious role on protein function predicted by bioinformatics tools.

a Sequence types of colistin-resistant *K. pneumoniae.*

b Genes analyzed in the study are included in the table, and associated mutations identified in a respective gene are given in the brackets.

In this study, *in vitro* antimicrobial susceptibility profiling and WHONET analysis categorized four *K. pneumoniae* isolate as pan drug-resistant and six as extensively drug-resistant (XDR) as per the definition by Magiorakos et al. (2012). Additionally, these isolates fall to the category of difficult-to-treat Gram-negative infections, where resistance to aminoglycosides emphasizes the difficulty of choosing salvage antibiotics for clinical containment (Kadri et al., 2018). The mortality in the present study was 36.4%, where only 7 out of 11 patients could recover. Three of the four patients deceased were infected with pan-drug resistant (PDR) *K. pneumoniae*. Among the 11 study isolates, positive amplification of *bla_NDM-1_* and *bla_OXA-48_* carbapenemase genes was found in 8 (73%) and 5 (45%) of the isolates, and two isolates were positive for both *bla_NDM-1_* and *bla_OXA-48_*. Colistin-resistant *K. pneumoniae* with PDR and XDR phenotype co-producing *bla_NDM-1_* and *bla_OXA-48_* carbapenamases have been reported to cause severe nosocomial infections in several countries (Guducuoglu et al., 2018; Moubareck et al., 2018; Haller et al., 2019).

In *K. pneumoniae*, the positively charged groups 4-amino-4-deoxy-L-arabinose (L-Ara4N) and phosphoethanolamine (PEtN) mediate the covalent modifications of lipid A moiety of LPS reducing the net negative charge and subsequently binding affinity of colistin (Tamayo et al., 2005a). Mutations in TCRSs and/or genetic alteration in the *mgrB* gene, the negative regulator of *phoP*/*phoQ* TCRS can cause constitutive expression of the *pmrHFIJKLM* and *pmrCAB* operons transferring the L-Ara4N and PEtN respectively, to lipid A of the cell membrane (Tamayo et al., 2005b). Along with six potentially deleterious mutations in *pmrB* gene, a deletion of 12 nucleotides resulting in the omission of four amino acids in the phospho-transfer domain of *phoQ* gene was observed in *K. pneumoniae* MRK5 strain tolerating 128 μg/ml of colistin. Marina et al. also reported similar deletions in the *phoQ* gene reducing the colistin susceptibility (Marina et al., 2001). MRK7 strain also demonstrated a MIC of 128 μg/ml for colistin having potentially deleterious substitution (H267P) in *pmrB* gene, underlining its significance. L30Q substitution in *phoQ* gene of MRK8 was similar to the earlier report (Cheng et al., 2015). The L30Q substitution in the hydrophobic domain (constituting 17–44 amino acid residues) of *phoQ* influences the protein conformation and oligomer stability potentially changing the phosphate transfer and the phosphatase ability (Goldberg et al., 2010). In two of *K. pneumoniae* isolates (MRK1 and MRK4), point mutation A170G was detected in the *pmrA* gene resulting in amino acid change (E57G) potentially deleterious while R256G in *pmrB* of MRK7 predicted not to cause any functional change in the protein function (Pragasam et al., 2017). The novel G250C potentially deleterious substitution in the *pmrB* gene was observed in three of *K. pneumoniae* isolates (MRK1, MRK4, and MRK5) that exhibited high MIC (≥128 μg/ml) for colistin. Several other deleterious substitutions in the *pmrB* (D150V, A252G, L237R, H267P, R315P, and Q331H) genes with their scores in the intolerant and potentially intolerant range are being reported here for the first time. These amino acid substitutions (L237R, A252G, H267P, R315P, Q331H, except D150V) were observed in a single isolate (MRK5) along with a four amino acid deletion in *phoQ* gene, emphasizing the need for further evaluation for their role in colistin resistance. Predicted deleterious substitution *pmrA*-Q140L (found in all 11 test isolates) and *pmrB* R256G (present in MRK7) have been previously reported in colistin-resistant as well as susceptible isolates negating its decisive role in colistin resistance (Pitt et al., 2018). However, the other mutations observed here, L30Q and A351D substitutions in the *phoQ* gene of MRK8 and MRK11 strains, respectively, and G53S in the *pmrA* gene of MRK10 and D150V and H267P in the *pmrB* gene of the MRK2 and MRK7 strain, respectively, may impart resistance in the absence of any other contributing factor.

The *mgrB* gene-mediated inactivation of the *phoP/phoQ* TCRS has been extensively reported to play a prominent role in polymyxin resistance in *K. pneumoniae* (Cannatelli et al., 2014b; Poirel et al., 2017). Three of 11 *K. pneumoniae* isolates MRK1, MRK3 and MRK4 had insertion inactivation of *mgrB* gene disrupting the translation of a functional protein, along with mutations in *pmrA* and *pmrB* genes showing high tolerance to colistin (MIC ≥128 μg/ml). The role of IS elements (IS*5*-like, IS*1F* and IS*Kpn14*, IS*Kpn13*, and IS*10R*) in *mgrB* inactivation that inhibits *phoQ* phosphorylation resulting in increased expression of *pmrHFIJKLM* mRNA and leading to reduced colistin susceptibility in *K. pneumoniae* have been extensively demonstrated (Cannatelli et al., 2014a; Poirel et al., 2017; Zhu et al., 2019). *K. pneumoniae* MRK1 and MRK4 were interrupted by an IS*1*-like transposase elements. The inactivation of *mgrB* protein with a similar IS*1*-like element interrupting at the same nucleotide position (117–118) was observed in *K. pneumoniae* recovered from a dead broiler from Iran (Pishnian et al., 2019). The *mgrB* gene in *K. pneumoniae* MRK3 isolate was disrupted by 969 bp long IS*5*/IS*1182*-like element at a different position. IS*5*/IS*1182* fragment is present in various plasmids isolated from different Gram-negative bacteria (Hala et al., 2019). Cannatelli et al. (2013) and Poirel et al. (2015) have reported insertional inactivation of *mgrB* gene with a similar IS*5*-like insertion sequence at 74–75 nucleotide position; however, the IS*5*-like element reported in MRK3 isolate of this study showed a different interruption of the nucleotide sequence at position 86–87. To the best of our knowledge, this insertional inactivation of *mgrB* gene by the IS*5*/IS*1182* fragment has not been reported before. Isolate MRK7 had M23R substitution having SIFT score of 0 with low confidence, PROVEAN score of −3.121 depicting intolerance and is found to be neutral with PolyPhen-2 hence treated as neutral while MRK9 had C28G substitution also observed by Shankar et al. (2019b), which shows highly intolerant tendency (Table 4). The remaining seven isolates had an intact wild type *mgrB* gene. Mutations observed in this study in the *pmrB* (T157P) and *mgrB* (C28Y) gene have been reported in previous studies to be linked with reduced colistin susceptibility in *K. pneumoniae* (Cannatelli et al., 2014a, 2015; Jayol et al., 2014; Lomonaco et al., 2018; Mathur et al., 2018). Isolates harboring IS*Kpn26*-like and other IS elements disrupting *mgrB* also exhibited mutations in *pmrA* and *pmrB* genes (Table 4).

To establish a correlation between lipid A modifying operon (*pmrCAB* and *pmrHFIJKLM*) expression pattern and colistin resistance among the test isolates, the expression level of *pmrK* and *pmrC* genes was evaluated. All isolates showed an over-expressed *pmrC* gene in comparison to the *pmrK* gene, implying that PEtN-mediated LPS modification plays a significant role in conferring colistin resistance in tested isolates. Among colistin-resistant *K. pneumoniae* isolates, fold-change increase in expression of the *pmrC* gene was higher in 6/11 (54%) isolates that decreased significantly upon colistin treatment (*p* < 0.05), though overall in both the conditions that level of expression of *pmrC* was higher compared to colistin sensitive isolate. For three isolates, the expression in both colistin treated and untreated conditions did not differ significantly, while the expression of *pmrC* increased significantly upon colistin treatment in two isolates.

The basal expression level of *pmrK* gene in 6/11 (54%) colistin-resistant *K. pneumoniae* isolates was similar to colistin sensitive isolate and showed significantly higher expression in three isolates. The expression of *pmrK* increased significantly upon colistin treatment in 7/11 (63%) isolates, remained unaltered in 3/11 (27%) isolates and repressed in one isolate MRK4. Isolate MRK11 exhibited repressed expression of *pmrK* in untreated condition exhibited >30 fold increase in the expression of *pmrK* gene upon colistin treatment. This isolate harbored deleterious change in *phoQ* gene that upon activation may increase phosphorylation of *phoP* activating transcription of *pmrHFIJKLM* operon resulting in increased *pmrK* expression. A similar increase in the expression of *pmrC* gene as operon regulators has been reported previously (Cheng et al., 2015; Haeili et al., 2017). T157P substitution in *pmrB* (observed in MRK9 isolate) has been shown to contribute to the overexpression of *pmrCAB* and *pmrHFIJKLM* operons with the *pmrC* being highly overexpressed (170-fold) compared to *pmrK* (40-fold; Jayol et al., 2014). The majority of the tested isolates (8/11) harbored *pmrB* gene with several mutations possibly contributing to constitutive expression of *pmrA* gene that may preferentially activate *pmrCAB* promoter compared to *pmrHFIJKLM* resulting in increased expression of the *pmr*C gene in these isolates. Similar to previous reports, no direct correlation between colistin exposure and gene upregulation was observed (Can et al., 2018). A single mechanism may not explain the role of *pmrC* and *pmrK* in mediating colistin resistance in *K. pneumoniae*, as we observed diverse strain-specific mutations that may affect the expression of these operons differentially. Our data indicates discrete emergence of colistin resistance in clinical isolates through strain-specific pathways where multiple mechanisms might be involved in resistance development.

## Conclusion

All 11 colistin and carbapenem-resistant *K. pneumoniae* isolates under study were distinct (with nine different ST types) and present the emergence of discrete colistin resistance mechanisms. The role of *mgrB* gene as a hot-spot for insertion inactivation and its functional loss associated with colistin resistance was observed in three isolates. Multiple mutations in regulatory genes (*phoP*, *phoQ*, *pmrA*, *pmrB*, and *mgrB*) and their association in the expression pattern of LPS decorating operons raises the colistin MIC values. The 11 test isolates were negative for the mobilized colistin resistance genes *mcr*-*1-8*, however, the occurrence of other *mcr* gene variants not investigated here could not be excluded as a contributory factor towards increased colistin MIC values. The clinical and public health concerns in a background where the pipeline for new antibiotics is limited, demand implementation of antimicrobial stewardship and infection control measures to prevent the spread of resistant bacteria in the health care settings. The upshot of the exorbitant and lavish use of antibiotics has developed severe concern regarding multidrug-resistant bacteria, especially nosocomial pathogens that necessitates the rational use of colistin as a last-resort antibiotic.

## Data Availability Statement

The datasets presented in this study can be found in online repositories. The names of the repository/repositories and accession number(s) can be found in the article/Supplementary Material.

## Ethics Statement

The studies involving human participants were reviewed and approved by ICMR-National Institute of Pathology, New Delhi, India (IEC No: NIP-IEC/2/3/17/06) and VMMC and Safdarjung Hospital, New Delhi, India (IEC No: IEC/VMMC/SJH/Project/January/2018/1028). The patients/participants provided their written informed consent to participate in this study.

## Author Contributions

Conceptualization, methodology, writing – review and editing: MA, RS, and RG. Data curation: MA, GY, AS, and KU. Formal analysis: MA, GY, AS, KU, and MJ. Funding acquisition: MA and RS. Investigation: RS and RG. Validation: RS, RG, and MJ. Writing – original draft: MA and GY. All authors contributed to the article and approved the submitted version.

### Conflict of Interest

The authors declare that the research was conducted in the absence of any commercial or financial relationships that could be construed as a potential conflict of interest.

## Footnotes

^1^https://bigsdb.pasteur.fr/klebsiella/klebsiella.html
