# Supplementary material for: Colistin Resistance Among Multiple Sequence Types of Klebsiella pneumoniae Is Associated With Diverse Resistance Mechanisms: A Report From India
Source: Front Microbiol. 2021 Feb 22;12:609840. doi: 10.3389/fmicb.2021.609840 (PMC7937630; doi:10.3389/fmicb.2021.609840)
Supplement: Supplementary file 1 [file Table_1.pdf]

Supplementary Table S1: Oligonucleotide sequences used in the study to amplify specific genes of eleven colistin-resistant *K. pneumoniae* isolates.

Gene	Primer Name	Oligo Seq	Reference
*pho*P	*pho*P ext F	5’-GAGCTTCAGACTACTATCGA-3’	Jayol et al., 2014
	*pho*P ext R	5’-GGGAAGATATGCCGCAACAG-3’	
phoQ	*pho*Q ext F	5’-ATACCCACAGGACGTCATCA-3’	
	*pho*Q ext R	5’-CAGGTGTCTGACAGGGATTA-3’	
*pmr*A	K*pmr*A ext F	5'- CATTTCCGCGCACTGTCTGC-3'	
	K*pmr*A ext R	5'- CAGGTTTCAGTTGCAAACAG-3'	
*pmr*B	K*pmr*B ext F	5'- ACCTACGCGAAAAGATTGGC-3'	
	K*pmr*B ext R	5'- GATGAGGATAGCGCCCATGC-3'	
*mgr*B	*mgr*B ext F	5’-TTAAGAAGGCCGTGCTATCC-3’	Cannatelli et al., 2013
	*mgr*B ext R	5’-AAGGCGTTCATTCTACCACC-3’	
16S rRNA	RRF	5’-GGCGGACGGGTGAGTAATG-3’	Azam et al., 2016
	RRR	5’-CGATTACTAGCGATTCCGACTTC-3’	
CTX-M	CTX-MF	5´-SCVATGTGCAGYACCAGTAA- 3´	
	CTX-MR	5´-GCTGCCGGTYTTATCVCC- 3	
bla$_{TEM}$	TEMF	5´-ATGAGTATTCAACATTTCCGTGT- 3´	
	TEMR	5´-TTACCAATGCTTAATCAGTGAGG- 3´	
bla$_{OXA-48}$	O48 F3	5′-CCAATAGCTTGATCGCCCTC-3′	
	O48 B3	5′-CCATAATCGAAAGCATGTAGC-3′	
bla$_{NDM-1}$	NDM F3	5'-GCATAAGTCGCAATCCCCG-3'	
	NDM B3	5'-GGTTTGATCGTCAGGGATGG-3'	
bla$_{KPC1}$	KPC1F	5’-AGCCGTTACAGCCTCTGGAG-3’	Kothari et al., 2013
	KPC1R	5’- GATGGGATTGCGTCAGTTCAG-3’	
bla$_{KPC2}$	KPC2F	5’-CACTGTATCGCCGTCTAGTTC-3’	
	KPC2R	5’-TGTGCTTGTCATCCTTGTTAG-3’	
mcr-1	M1F	5′-CGGTCAGTCCGTTTGTTC-3′	Liu et al., 2016
	M1R	5′-CTTGGTCGGTCTGTAGGG-3′	
mcr-2	M2F	5’-TGTTGCTTGTGCCGATTGGA -3’	Xavier et al., 2016
	M2R	5’ –AGATGGTATTGTTGGTTGCTG- 3’	
mcr-3	M3F	5’-TTG GCACTGTATTTTGCATTT-3’	Yin et al., 2017
	M3R	5’-TTAACGAAATTGGCTGGAACA-3’	
mcr-4	M4F	5’-ATTGGGATAGTCGCCTTTTT-3’	Carattoli et al., 2017
	M4R	5’-TTACAGCCAGAATCATTATCA-3’	
mcr-5	M5F	5’-GTGAAACAGGTGATCGTGACTTACCG-3’	Chen et al., 2018
	M5R	5’-CGTGCTTTACACCGATC ATGTGCT-3’	
mcr-7	M7F	5’-TTATCAACCGCTGGGGACTG-3’	This Study
	M7R	5’-GCTTGATCTCGATGTTGGGCAC-3’	

mcr-8	M8F	5'-GAAACTGGTGGTTGTCGTCGTG-3'	
	M8R	5'-AGCACCTCAACACCTGTACG-3'	
RT*pmr*C	RT*pmr*C-F	5′-CTCTCGCCTCGTTCCTGAA-3′	
	RT*pmr*CR	5′-CGGAGTGGTGTCGAGGATA-3′	
RT*pmr*K	RT*pmr*KF	5′-GGTGTATGCGATTGGCACCTA-3′	
	RT*pmr*KR	5′-AGCAGCACGTAGCCCAGTAT-3′	
RTrpsl	RTrpslF	5′-CCGTGGCGGTCGTGTTAAAGA-3′	
	RTrpslR	5′-GCCGTACTTGGAGCGAGCCTG-3′	

References:

Azam, M., Jan, A.T., Haq, Q.M.R. (2016) . *bla*CTX-M-152, a novel variant of CTX-M-group-25, identified in a study performed on the prevalence of multidrug resistance among natural inhabitants of river Yamuna, India. *Frontiers in microbiology,* 7, 176. doi: 10.3389/fmicb.2016.00176.

Cannatelli, A., D'Andrea, M.M., Giani, T., Di Pilato, V., Arena, F., Ambretti, S., Gaibani, P. and Rossolini, G.M. (2013). In vivo emergence of colistin resistance in *Klebsiella pneumoniae* producing KPC-type carbapenemases mediated by insertional inactivation of the PhoQ/PhoP *mgr*B regulator. *Antimicrob Agents Chemother*. 57(11):5521-5526. doi:10.1128/AAC.01480-13.

Carattoli, A., Villa, L., Feudi, C., Curcio, L., Orsini, S., Luppi, A., Pezzotti, G. and Magistrali, C.F. (2017) Novel plasmid-mediated colistin resistance *mcr-4* gene in *Salmonella* and *Escherichia coli*, Italy 2013, Spain and Belgium, 2015 to 2016. *Euro Surveill*. 2017;22(31):30589. doi:10.2807/1560-7917.ES.2017.22.31.30589.

Chen, L., Zhang, J., Wang, J., Butaye, P., Kelly, P., Li, M., Yang, F., Gong, J., Yassin, A.K., Guo, W. and Li, J. (2018) Newly identified colistin resistance genes, mcr-4 and mcr-5, from upper and lower alimentary tract of pigs and poultry in China. *PLoS ONE* 13(3): e0193957. doi.org/ 10.1371/journal.pone.0193957.

Jayol, A., Poirel, L., Brink, A., Villegas, M.V., Yilmaz, M., Nordmann, P. (2014) Resistance to colistin associated to a single amino acid change in protein PmrB among *Klebsiella pneumoniae* of worldwide origin. *Antimicrob Agents Chemother*; 58:4762–4766. doi: 10.1128/AAC.00084-14.

Kothari, C., Gaind, R., Singh, L.C., Sinha, A., Kumari, V., Arya, S., Chellani, H., Saxena, S. and Deb, M. (2013) Community acquisition of β-lactamase producing *Enterobacteriaceae* in neonatal gut. *BMC Microbiol.* 17; 13:136. doi: 10.1186/1471-2180-13-136.

Liu, Y.Y., Wang, Y., Walsh, T.R., Yi, L.X., Zhang, R., Spencer, J., Doi, Y., Tian, G., Dong, B., Huang, X. and Yu, L.F. (2016) Emergence of plasmid-mediated colistin resistance mechanism *MCR-1* in animals and human beings in China: a microbiological and molecular biological study. *Lancet Infect Dis.* 16(2):161-168. doi: 10.1016/S1473-3099(15)00424-7.

Xavier, B.B., Lammens, C., Ruhal, R., Kumar-Singh, S., Butaye, P., Goossens, H. and Malhotra-Kumar, S., (2016). Identification of a novel plasmid-mediated colistin-resistance gene, *mcr2,* in *Escherichia coli*, Belgium, June 2016. Euro Surveill. 21(27):pii=30280. doi: org/10.2807/1560-7917.ES.2016.21.27.30280.

Yin, W., Li, H., Shen, Y., Liu, Z., Wang, S., Shen, Z., Zhang, R., Walsh, T.R., Shen, J. and Wang, Y. (2017) Novel plasmid-mediated colistin resistance gene mcr-3 in *Escherichia coli*. *mBio* 8:e00543-17. doi.org/10.1128/mBio .00543-17.
