# Supplementary material for: Colistin Resistance Among Multiple Sequence Types of Klebsiella pneumoniae Is Associated With Diverse Resistance Mechanisms: A Report From India
Source: Front Microbiol. 2021 Feb 22;12:609840. doi: 10.3389/fmicb.2021.609840 (PMC7937630; doi:10.3389/fmicb.2021.609840)
Supplement: Supplementary file 2 [file Table_2.pdf]

List of amino acid substitutions observed in *phoP, phoQ, mgrB, pmrA* and *pmrB* genes among colistin resistant *K. pneumoniae* isolates.

	Gene	Mutation	SIFT (Score)	PROVEAN (score)	PolyPhen-2 (score)	Mutation Type[# $]
Common to MRK1 – MRK11 isolates	mgrB	Nil	NA	NA	NA	NA
	phoP	K34R	TOLERATED (score 0.55)	Neutral (-0.660)	BENIGN (score 0.000)	Neutral
	phoQ	K64R	AFFECT PROTEIN FUNCTION* (score 0.01)	Neutral (score -0.274)	BENIGN (score 0.006)	Neutral
		K92Q	AFFECT PROTEIN FUNCTION* (score 0.04)	Neutral (score 0.020)	BENIGN (score 0.026)	Neutral
		T106A	TOLERATED (score 0.06)	Neutral (score 0.143)	BENIGN (score 0.000)	Neutral
		D112E	TOLERATED (score 0.76)	Neutral (score 0.154)	BENIGN (score 0.000)	Neutral
		V139I	TOLERATED (score 0.76)	Neutral (-0.129)	BENIGN (score 0.000)	Neutral
		F163L	AFFECT PROTEIN FUNCTION* (score 0.04)	Neutral (score 0.555)	BENIGN (score 0.000)	Neutral
		I196V	TOLERATED (score 0.60)	Neutral (score 0.077)	BENIGN (score 0.001)	Neutral
		S372T	TOLERATED (score 0.31)	Neutral (score 1.136)	BENIGN (score 0.004)	Neutral
		P424Q	TOLERATED (score 0.57)	Neutral (score 0.069)	BENIGN (score 0.001)	Neutral
		L482Q	TOLERATED (score 0.25)	Neutral (score 1.731)	BENIGN (score 0.010)	Neutral
		E487Q	AFFECT PROTEIN FUNCTION* (score 0.00)	Neutral (score -0.387)	POSSIBLY DAMAGING (score 0.881)	Neutral
	pmrA	A64S	TOLERATED (score 0.77)	Neutral (score 0.346)	BENIGN (score 0.001)	Neutral
		D131N	TOLERATED (score 1.00)	Neutral (score 0.293)	BENIGN (score 0.001)	Neutral
		Q140L	TOLERATED (score 0.56)	Deleterious (score -4.151)	PROBABLY DAMAGING (score 0.996)	Potentially Deleterious
		D199E	TOLERATED (score 1.00)	Neutral (score 1.327)	POSSIBLY DAMAGING (score 0.489)	Neutral
		H219N	AFFECT PROTEIN FUNCTION* (score 0.00)	Neutral (score 0.712)	BENIGN (score 0.000)	Neutral
	pmrB	N8T	TOLERATED (score 0.71)	Neutral (score 0.305)	BENIGN (score 0.000)	Neutral
		S105N	TOLERATED (score 0.61)	Neutral (score -0.379)	BENIGN (score 0.102)	Neutral
		T228A	TOLERATED (score 0.50)	Neutral (score 0.400)	BENIGN (score 0.097)	Neutral

List of amino acid substitutions observed in *phoP, phoQ, mgrB, pmrA* and *pmrB* genes among colistin resistant *K. pneumoniae* isolates.

		E232Q<br>V242I (except K1 and 5)<br>S244N<br>Q272E<br>R356Q	TOLERATED (score 0.49)<br>TOLERATED (score 0.12)<br>TOLERATED (score 0.50)<br>TOLERATED (score 1.00)<br>TOLERATED (score 0.15)	Neutral (score 0.649)<br>Neutral (score -0.187)<br>Neutral (score -0.901)<br>Neutral (score 1.552)<br>Neutral (score 0.290)	BENIGN (score 0.000)<br>BENIGN (score 0.385)<br>BENIGN (score 0.000)<br>BENIGN (score 0.000)<br>BENIGN (score 0.322)	Neutral<br>Neutral<br>Neutral<br>Neutral<br>Neutral
**MRK1**	mgrB	IS element	NA	NA	NA	NA
	pmrA	E57G	TOLERATED (score 0.33.)	Neutral (score -1.934)	BENIGN (score 0.006)	Neutral
	pmrB	V242M<br>G250C<br>A252G	TOLERATED (score 0.12)<br>AFFECT PROTEIN FUNCTION (score 0.01)<br>TOLERATED (score 0.40)	Neutral (score -0.106)<br>Deleterious (score -7.908)<br>Deleterious (score -3.368)	BENIGN (score 0.385)<br>PROBABLY DAMAGING (score 1.000)<br>PROBABLY DAMAGING (score 0.999)	Neutral<br>Potentially Deleterious<br>Potentially Deleterious
**MRK2**	mgrB	No mutation	NA	NA	NA	NA
	phoQ	H235Q<br>T244A	TOLERATED (score 0.1)<br>TOLERATED (score 0.08)	Neutral (score 1.371)<br>Neutral (score -0.308)	BENIGN (score 0.015)<br>BENIGN (score 0.252)	Neutral<br>Neutral
	pmrB	D150V	AFFECT PROTEIN FUNCTION (score 0.00)	Deleterious (score -8.513)	PROBABLY DAMAGING (score 1.000)	Potentially Deleterious
**MRK3**	mgrB	IS element	NA	NA	NA	NA
	pmrB	A246T<br>L332M	TOLERATED (score 0.20)<br>TOLERATED (score 0.15)	Neutral (score -0.970)<br>Neutral (score -1.495)	BENIGN (score 0.000)<br>PROBABLY	Neutral<br>Neutral

List of amino acid substitutions observed in *phoP, phoQ, mgrB, pmrA* and *pmrB* genes among colistin resistant *K. pneumoniae* isolates.

					DAMAGING (score 1.000)	
**MRK4**	mgrB	IS element	NA	NA	NA	NA
	pmrA	E57G	TOLERATED (score 0.33)	Neutral (score -1.934)	BENIGN (score 0.006)	Neutral
	pmrB	G250C	AFFECT PROTEIN FUNCTION (score 0.01)	Deleterious (score -7.908)	PROBABLY DAMAGING (score 1.000)	Potentially Deleterious
**MRK5**	mgrB	No mutation	NA	NA	NA	NA
	phoQ	Del (87-90)		score -8.139,-9.132, -12.668,-11.814 (Avg.; -10.43825)		Deleterious
	pmrA	A217V	AFFECT PROTEIN FUNCTION* (score 0.03)	Neutral (score -0.787)	BENIGN (score 0.000)	Neutral
	pmrB	L237R<br>V242M<br>G250C<br>A252G<br>H267P<br>L284P<br>R315P<br>A330V	TOLERATED (score 0.24)<br>TOLERATED (score 0.12)<br>AFFECT PROTEIN FUNCTION (score 0.01)<br>TOLERATED (score 0.40<br>AFFECT PROTEIN FUNCTION (score 0.04)<br>TOLERATED (score 0.05)<br>AFFECT PROTEIN FUNCTION (score 0.04)<br>TOLERATED (score 0.05)	Deleterious(score -3.395)<br>Neutral (score -0.106)<br>Deleterious (score -7.908)<br>Deleterious (score -3.368)<br>Deleterious (score -4.767)<br>Neutral (score 2.297)<br>Deleterious (score -5.393)<br>Neutral (score -0.176)	PROBABLY DAMAGING (score 1.000)<br>BENIGN (score 0.385)<br>PROBABLY DAMAGING (score 1.000)<br>PROBABLY DAMAGING (score 0.999)<br>PROBABLY DAMAGING (score 0.999)<br>BENIGN (score 0.000)<br>PROBABLY DAMAGING (score 1.00)<br>BENIGN (score 0.097)	Potentially Deleterious<br>Neutral<br>Potentially Deleterious<br>Potentially Deleterious<br>Potentially Deleterious<br>Neutral<br>Potentially Deleterious<br>Neutral

List of amino acid substitutions observed in *phoP, phoQ, mgrB, pmrA* and *pmrB* genes among colistin resistant *K. pneumoniae* isolates.

		Q331H<br><br>L332M	AFFECT PROTEIN FUNCTION (score 0.05)<br>TOLERATED (score 0.06)	Deleterious (score -3.237)<br><br>Neutral (score -1.495)	PROBABLY DAMAGING (score 1.000)<br>PROBABLY DAMAGING (score 1.000)	Potentially Deleterious<br>Neutral
**MRK6**	mgrB	No mutation	NA	NA	NA	NA
	phoQ	DEL (263-64)		score -8.710,-10.177 (AVG: 9.4435)		Potentially Deleterious
	pmrB	A246T<br>A252G	TOLERATED (score 0.20)<br>TOLERATED (score 0.40)	Neutral (score -0.970)<br>Deleterious (score -3.368)	BENIGN (score 0.178)<br>PROBABLY DAMAGING (score 0.999)	Neutral<br>Potentially Deleterious
**MRK7**	mgrB	M23R	AFFECT PROTEIN FUNCTION* (score 0.00)	Deleterious (score -3.121)	BENIGN (score 0.001)	Neutral
	pmrB	E245K<br><br>R256G<br><br>H267P	AFFECT PROTEIN FUNCTION (score 0.05)<br>TOLERATED (score 0.28)<br><br>TOLERATED (score 0.21)	Neutral (score -1.245)<br><br>Deleterious (score -5.484)<br><br>Deleterious (score -4.767)	BENIGN (score 0.270)<br><br>PROBABLY DAMAGING (score 0.975)<br>PROBABLY DAMAGING (score 0.999)	Neutral<br><br>Potentially Deleterious<br>Potentially Deleterious
**MRK8**	mgrB	No mutation	NA	NA	NA	NA
	phoQ	L30Q<br><br>H234Y	AFFECT PROTEIN FUNCTION* (score 0.00)<br>TOLERATED (score 0.11)	Deleterious (score -4.339)<br><br>Neutral (score -1.259)	PROBABLY DAMAGING (score 1.000)<br>PROBABLY DAMAGING (score 0.982)	Potentially Deleterious<br>Neutral

List of amino acid substitutions observed in *phoP, phoQ, mgrB, pmrA* and *pmrB* genes among colistin resistant *K. pneumoniae* isolates.

**MRK9**	mgrB	C28G	AFFECT PROTEIN FUNCTION* (score 0.00)	Deleterious (score -12.00)	POSSIBLY DAMAGING (score 0.915)	Potentially Deleterious
	phoP	T151A	AFFECT PROTEIN FUNCTION* (score 0.00)	Deleterious (score -4.805)	PROBABLY DAMAGING (score 1.000)	Potentially Deleterious
	pmrB	T157P<br>A246T	AFFECT PROTEIN FUNCTION (score 0.02)<br>TOLERATED (score 0.20)	Deleterious (score -5.787)<br>Neutral (score -0.970)	BENIGN (score 0.000)	Potentially Deleterious<br>Neutral
**MRK10**	mgrB	No mutation	NA	NA	NA	NA
	pmrA	G53S<br>M66I	TOLERATED (score 0.97)<br>TOLERATED (score 0.62)	Deleterious (score -5.069)<br>Neutral (score -0.624)	PROBABLY DAMAGING (score 1.000)<br>BENIGN (score 0.000)	Potentially Deleterious<br>Neutral
**MRK11**	mgrB	No mutation	NA	NA	NA	NA
	phoQ	A351D	TOLERATED (score 0.26)	Deleterious (score -4.223)	PROBABLY DAMAGING (score 1.000)	Potentially Deleterious

Criteria used by different Software's to designate amino acid substitution as Neutral or Deleterious:
SIFT: The amino acid substitution is predicted to affect protein function (intolerant) if the score is <=0.05, and tolerant if the score is >0.05.
PolyPhen-2: default threshold value 50%
PROVEAN: default score threshold -2.5.

*Denotes Low Confidence Interval.
[#]The mutations found to be deleterious by at least two of the three analysis software's were considered, while mutations with low confidence interval were treated as neutral.

List of amino acid substitutions observed in *phoP, phoQ, mgrB, pmrA* and *pmrB* genes among colistin resistant *K. pneumoniae* isolates.

[$] Neutral: any mutation predicted to not affecting the protein function and identified as tolerated (SIFT), neutral (PROVEAN) or benign (PolyPhen-2).<br>Potentially Deleterious: Any mutation predicted to affect the protein function and identified as affecting protein function (SIFT), deleterious (PROVEAN) and damaging (PolyPhen-2).
**Note-** The neutral or potentially deleterious nature of the amino acid substitutions was predicted by the bioinformatics tools. Experimental validation for the role of mutation in contributing colistin resistance was not performed.
